# Development of a midwifery regulatory environment index using data from the Global Midwives’ Associations map survey

**DOI:** 10.1186/s12913-025-12694-w

**Published:** 2025-05-20

**Authors:** Emma Virginia Clark, Marianna LaNoue, Kate Clouse, Alexandra Zuber, Jeremy Neal

**Affiliations:** 1 School of Nursing, Vanderbilt University, 461 21St Ave. S., Nashville, TN 37240 USA; 2 Ata Health Strategies LLC, 1537 D Street NE, Washington, D.C., 20002 USA

**Keywords:** Model selection, Model comparison, Midwifery, Regulation, Regulatory environment, Secondary data analysis

## Abstract

**Background:**

Global policymakers have proposed strengthening midwifery regulation to improve access to and quality of care provided by midwives, thereby enhancing maternal healthcare delivery and outcomes. However, quantifying ‘midwifery regulatory environments’ as a construct across countries has been difficult, limiting our ability to evaluate relationships between regulatory environments and key outcomes and hindering actionable steps toward improvement. The Global Midwives’ Associations map survey includes data across five domains of regulation (overarching regulatory policy and legislation; education and qualification; licensure; registration/re-licensure; and scope and conduct of practice). We aimed to use these data to develop a composite index that represents the midwifery regulatory environment in the countries that participated in the survey.

**Methods:**

To develop our composite Midwifery Regulatory Environment (MRE) Index, we analyzed data from 115 countries in the Global Midwives’ Associations map survey. We identified five different possible scoring characterizations for thirteen regulatory items. Four characterizations used continuous or categorical cumulative scoring and one used multiple individual components scoring. We compared these characterizations using Clarke’s test and descriptive model fit metrics to identify the best fit and performance for three outcomes: maternal mortality ratio, low birthweight prevalence, and stillbirth rate.

**Results:**

The Aggregated Domain Scoring method, which assigns one point for each of the five essential regulatory domains with activity (possible score range: 0–5), was the best fit and performing characterization for maternal mortality ratio and stillbirth outcomes. The Any-or-None Scoring method, which assigns one point per survey item with regulatory activity (possible score range: 0–13), best fit low birthweight prevalence.

**Conclusions:**

Our study demonstrates that developing composite characterizations of complex constructs, as exemplified by MRE Index development, can enhance the usability of existing global health datasets. Additionally, it highlights how employing model fit prediction provides a transparent, replicable, and accessible approach for identifying the optimal characterization of the construct based on a specific outcome. Specifically, we found that different characterizations for the MRE Index are preferred for different maternal health outcomes. The MRE Index we have developed stands as a valuable tool for future research exploring relationships between midwifery regulation and maternal health outcomes.

**Supplementary Information:**

The online version contains supplementary material available at 10.1186/s12913-025-12694-w.

## Background

The 2014 Lancet Series on Midwifery provided a comprehensive review of the role and potential contributions of midwives, strengthening the evidence for the broader use of midwives as an essential and cost-efficient strategy for improving maternal health outcomes [1]. Modeling studies indicate that universal coverage of a set of life-saving high-impact interventions, including family planning, preconception, antepartum, intrapartum, and postpartum care, that can be delivered by midwives could prevent 67% of all maternal deaths [2]. However, the midwifery workforce faces numerous obstacles in many countries, hindering their capacity to achieve this end. These barriers include unclear roles, inconsistent or limited scopes of practice, absent or limited continuing professional development programs, varying educational and clinical standards for entry to practice, and weak licensure and registration authorities and systems [3].

Strengthening midwifery regulation has been proposed by policymakers as a means of overcoming existing barriers and improving maternal health care in low- and middle-income countries (LMICs) [4]. Healthcare professional regulation establishes mechanisms and standards that define the roles of different healthcare professional groups [5]. Midwifery regulation, specifically, refers to the legislation and resultant criteria and procedures that determine the qualifications of midwives, delineate their scope of practice, and differentiate them from non-midwives [6]. Regulation is intended to support midwives to work autonomously within their full scope of practice to assure standards of maternity care and maternal and newborn health outcomes, and is a priority component of addressing health workforce challenges [7]. There are five major domains of midwifery regulation: overarching regulatory policy and legislation, education and qualification, licensure, registration/re-licensure, and scope and conduct of practice [6, 8–10]. A strong midwifery regulatory environment is one wherein the profession is recognized to be a distinct profession under legislation, having its own regulatory body or track within a broader regulatory body. This regulatory body must have statutory authority and be capable of implementing and overseeing activities across the major domains of regulation (1) to ensure prescribed educational standards are met by all midwives; (2) to establish and maintain transparent standards and processes for entering and remaining in practice; (3) to ensure the defined midwifery scope of practice aligns with the International Confederation of Midwives’ (ICM) definition of a midwife; and, (4) to establish transparent procedures for addressing complaints and to administer discipline, as needed [6, 8–10]. A weak regulatory environment is one where most or all of these structures, mechanisms, and/or activities are absent, curtailed, or non-functional.

Despite delineation of the components and functions of midwifery regulation [8–10], few attempts have been made to measure and quantify regulatory environments in a consistent way across countries. Additionally, there is a lack of research regarding the influence of regulatory environments on maternal health outcomes that limits actionability toward ICM and the World Health Organization (WHO) calls for improved midwifery regulation. This underscores the importance for additional research to inform and justify investments in transformative regulatory initiatives, particularly research that establishes a clear link between regulatory efforts and measurable outcomes.

The Global Midwives’ Associations map survey is a high-quality dataset available to the public, containing information on midwifery associations, education, leadership, and regulation across more than a hundred countries [11]. This is a crucial resource for research on global midwifery regulation. When paired with other aggregated databases such as the Global Health Data Exchange [12] and WHO Global Health Observatory [13], datasets like the Global Midwives’ Association map survey enable research teams to cost-effectively explore new questions using existing, high-quality data from diverse national and global sources [14, 15]. Analyzing secondary data is a widely recognized method for developing evidence required in global health initiatives, policymaking, and investments. For example, use of Demographic Health Survey data was instrumental in recommending future funding allocation for marginalized groups in Nigeria to achieve universal maternal health coverage [16]. Similarly, examining maternal death audit data has helped identify obstacles to implementing postpartum hemorrhage recommendations in Kenya [17].

Maximizing the usefulness of existing datasets frequently requires aggregating two or more variables that represent multiple dimensions of a particular domain or construct into a unified composite measure in order to effectively capture complex health system and service delivery contexts [15]. This can increase reliability by decreasing reliance on a single indicator [18]. However, given the potential impact on policy and programming decision-making, researchers must be conscientious in defining and operationalizing health system components to prevent oversimplification or misrepresentation in composite scores [18]. Given this, a prerequisite step before using data from the Global Midwives’ Associations map survey to evaluate the relationship between midwifery regulatory environments and maternal health outcomes is to aggregate responses from regulatory-focused survey questions into a composite regulatory measure that encompasses the five regulatory domains. Our primary aim with this study is to use responses from the Global Midwives’ Associations map survey to develop a composite index that represents the midwifery regulatory environment in the countries that participated in the survey.

## Methods

We conducted secondary data analysis on the multi-country Global Midwives’ Associations map survey dataset, which includes questions associated with the regulatory aspects of midwifery. The ICM, in charge of administering the survey, derived the regulatory section from their Global Standards for Midwives [6], which were established through expert consensus by a multi-member ICM-appointed taskforce with input from regulators and ICM member associations [19]. We identified five different characterizations of scoring regulatory indicators to create a composite score we called the ‘Midwifery Regulatory Environment (MRE) Index’. We assessed model fit and performance in prediction of outcomes for each of the five characterizations to determine the most appropriate characterization for use in evaluating relationships between the MRE Index and maternal health outcomes in future research. This study was approved by the Vanderbilt University Medical Center Institutional Review Board.

### Conceptual framework

**Fig. 1:**
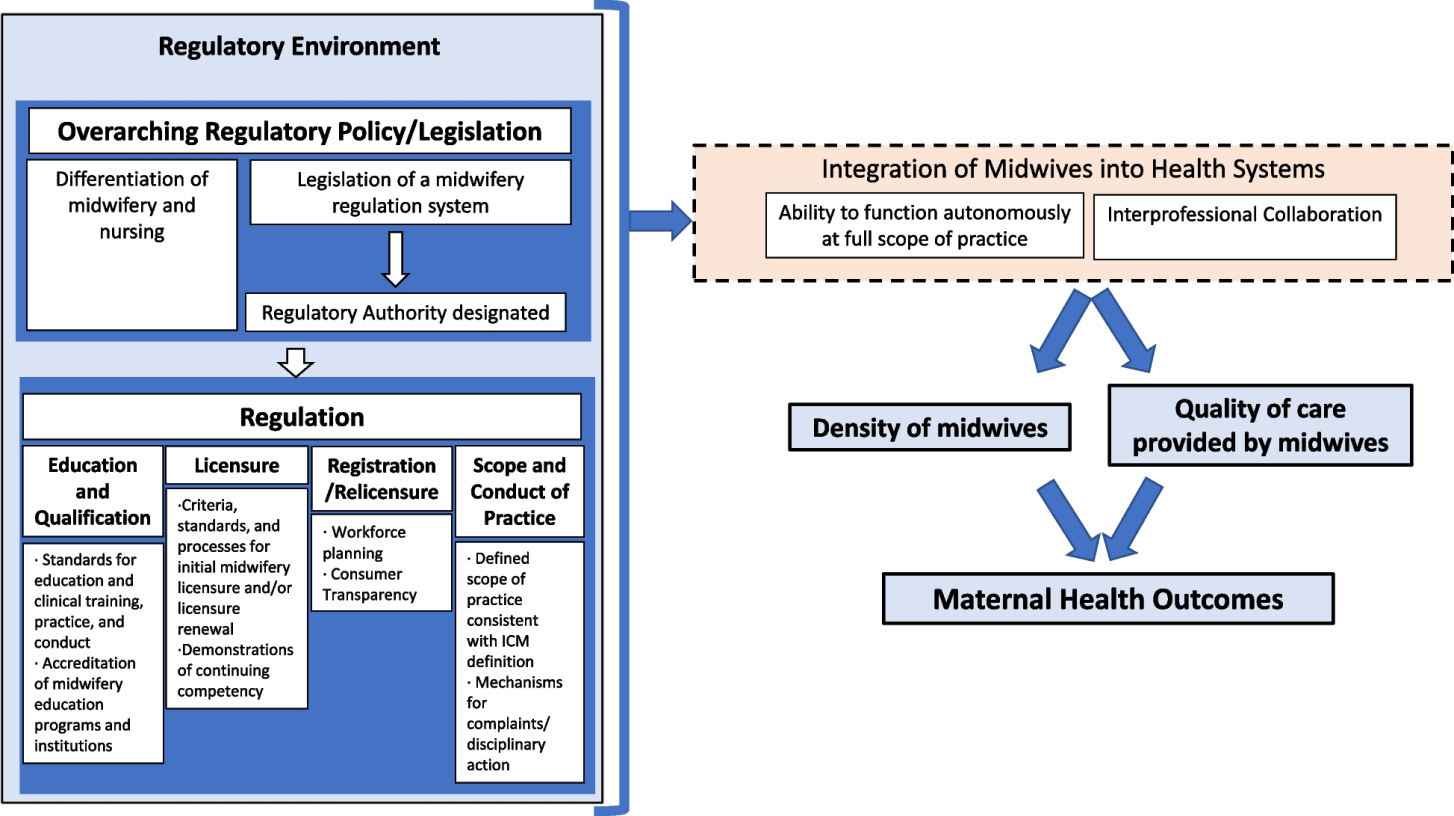
Conceptual framework

Before analysis, we developed a conceptual framework depicting the essential MRE domains from the ICM Global Standards for Regulation [6] (i.e., *overarching regulatory policy and legislation*; *education and qualification*; *licensure*; *registration/re-licensure*; and *scope and conduct of practice*) and their key components as described by several research teams [8–10] (Fig. 1). This framework posits that MRE impacts the highest-level *maternal health outcomes* such as maternal death (measured by maternal mortality ratio [MMR]), mediated by the extent to which midwives are integrated into the health system. *Integration of midwives* is operationalized as (1) the *density of midwives* (a proximal indicator for access to midwifery care), and (2) the *quality of care provided by midwives* (including high-level indicators such as LBW prevalence and stillbirth rate) [20, 21]. This framework guided components included in MRE Index characterization and variable selection in modeling MRE scoring characterization performance.

### Data sources and variables

The Global Midwives’ Associations map survey was developed by ICM and the United Nations Population Fund (UNFPA) and sent to all 142 ICM member associations in October of 2019 [19]. Countries with no ICM member association were invited to participate through UNFPA and WHO regional offices. The survey included four sub-surveys: association, education, leadership data, and regulation and was available in English, Russian, Spanish, and French. Respondents were country midwifery associations, with support from the UNFPA Country Office and relevant government authorities/organization, and data validation was conducted by an analysis team contracted by ICM and country government officials [19]. One hundred-fifteen member countries submitted responses to the regulatory subsection.

For our dependent variables, we used publicly available data from the United Nations Maternal Mortality Estimation Inter-Agency Group for a country’s MMR, defined as the number of female deaths from any cause related to pregnancy or its management, excluding accidental causes, per 100,000 live births in a given year [22]. The United Nations Children’s Fund (UNICEF)-WHO 2019 low birthweight (LBW) database provided data for the LBW prevalence outcome, defined as the percentage of newborns at birth that weight less than 2,500 g regardless of gestational age [23]. We used United Nations Inter-Agency Group for Child Mortality Estimation Core Stillbirth Estimation Group for the stillbirth outcome, defined as the number of babies born without signs of life at or after 28 weeks of gestation, per 1,000 live births [24]. Each dependent variable was treated as continuous, and transformations were undertaken when necessary. We incorporated the same set of four covariates in all models to adjust for economic and socioeconomic variations between countries. We obtained covariate data for our modeling from several publicly available datasets, including the United Nation Development Program’s (UNDP) Human Development Index (HDI) [25] and WHO’s Global Health Expenditure [26] databases, as well as the World Bank’s Gini Index [27] and income group [28] databases. Two of these covariates (country health spending in US dollars and the Gini Index, which measures the extent to which the distribution of income or consumption among individuals or households within an economy deviates from a perfectly equal distribution on a scale of 0 [perfect equity]− 1 [perfect inequality] [27]) were continuous variables. The other two covariates were categorical with four categories each. HDI is a composite measure of average achievement in three key dimensions of human life: a long and healthy life measured by life expectancy at birth; education, measured by mean years of schooling and expected years of schooling; and a decent standard of living, measured by gross national income per capita in Purchasing Power Parity (PPP) international dollars. HDI is reported on a scale from 0 to 1.00 and categorized by UNDP as Low (HDI < 0.550), Medium (HDI between 0.550 and 0.699), High (HDI between 0.700 and 0.799) and Very High (HDI ≥ 0.800) [25]. A country’s income group is assigned annually by the World Bank based on the gross national income (GNI) per capita of the previous year, expressed in United States dollars. In 2022, countries were classified as low income (GNI per capita less than $1,085), lower-middle income (GNI per capita $1,086–4,255), upper-middle income (GNI per capita $4,256–13,205), and high income (GNI per capita greater than $13,205).

### Alignment of data with conceptual framework

The regulatory subsection of the Global Midwives’ Associations map survey consists of 38 questions identified by ICM and the State of the World’s Midwifery Core Group [11]. We began by selecting questions appropriate for inclusion as items in the MRE Index. Of the 38 regulatory subsection questions, eight were excluded because they were open-ended and asked for free-text information (e.g., the source of or link to data provided in another question), and another eight were excluded as they did not directly relate to at least one regulatory domain (e.g., number of non-practicing midwives). We consolidated five questions on individual components of family planning scope of practice into one family planning scope of practice item. Likewise, we consolidated seven questions on individual components of basic emergency obstetric and newborn care (BEmONC) into a specific BEmONC scope of practice item. Nine of the original regulatory subsection questions were included without change and a final question about the frequency of re-licensure (question RS17) was included after answer free-text options were re-coded into a yes/no format (i.e., “Never” or “0” coded as no, all other numeric answers coded as yes). This resulted in a total of 12 items representing 22 questions to be used in composite MRE Index development (Table 1).

**Table 1:** ICM Global Midwives’ Associations map survey questions included in composite MRE index development

Item number	ICM question number	Question	Answer choices	Assigned domain
1	Regulation Sub-Survey (RS) 3	Is there legislation in your country which recognizes midwifery as a profession that is distinct from nursing?	Y/N	Overarching Regulatory Policy and Legislation
2	RS4	Is there legislation through which midwives are regulated?	Y/N	Overarching Regulatory Policy and Legislation
3	RS6	Is there a regulation system for midwives in your country?	Y/N	Overarching Regulatory Policy and Legislation
4	RS12	If the regulatory authority regulates midwives and other professions, are there separate and distinct policies and processes?	Y/N	Overarching Regulatory Policy and Legislation
5	RS15	Is there a system for licensing midwives?	Y/N	Licensure
6	RS16	Is licensing compulsory before midwives start to practice in the country?	Y/N	Licensure
7	RS17	Are midwives required to relicense in order to practice?^a^	Y/N	Licensure
8	RS18	As a condition of re-licensing, are midwives required to provide evidence that they have participated in continuing professional development activities?	Y/N	Licensure
9	RS20	Is registration required to practice midwifery in the country?	Y/N	Registration/Re-licensure
10	RS21	How often does this organization update the register to add new midwives or remove those who have left the profession?	Daily/Monthly/Several times a year)/Annually/Less Often^b^	Registration/Re-licensure
11	RS27-RS33	Are midwives authorized to provide this BEmONC function:		Scope and Conduct of Practice
		• parenteral administration of antibiotics	Y/N	
		• administration of anticonvulsants	Y/N	
		• administration of oxytocics	Y/N	
		• manual removal of placenta	Y/N	
		• manual vacuum aspiration for retained products	Y/N	
		• assisted instrumental delivery by vacuum extractor	Y/N	
		• newborn resuscitation with mask	Y/N	
12	RS34-RS38	Are midwives in this country authorized to provide:		Scope and Conduct of Practice
		• contraceptive injection	Y/N	
		• contraceptive pill	Y/N	
		• intrauterine device	Y/N	
		• emergency contraception (morning after pill)	Y/N	
		• contraceptive implant	Y/N	
13	Education Sub-Survey (ES) 4	Does your country have a national curriculum for midwifery education, whether is direct entry or post-nursing?	Yes, and all schools follow it/Yes, and some schools follow it/No^c^	Education and Qualification

^a^Question rewritten and recoded from free-text question “ How often are midwives required to relicense in order to practice (in years)?”

^b^Scoring assigned as Daily = 4; Monthly = 3; Several times a year = 2; Annually = 1; Less Often = 0

^c^Scoring assigned as Yes, and all schools follow it = 2; Yes, and some schools follow it = 1; No = 0

Next, we associated each item with its respective regulatory domain, i.e., overarching regulatory policy and legislation; education and qualification; licensure; registration/re-licensure; scope and conduct of practice (Table 1). Since there were no education and qualification-domain related questions in the regulatory subsection, we included a question about the national curriculum for midwifery education from the education subsection of the survey for incorporation into the composite MRE Index [11]. The final set of 13 items (12 from the regulatory sub-section and 1 from the education sub-section) are listed in Table 1.

### Data screening and analysis

One hundred fifteen countries (80% of the 142 member countries) provided complete responses in the regulatory subsection of the Global Midwives’ Associations map survey as well as a complete response to the education subsection question, allowing for computation of composite MRE Index using each of the five characterizations described below. We excluded nine countries with missing data for one or more covariates (i.e., Health Spending, Gini Index, HDI, and/or income group) from analyses. MMR data were missing for three countries, while stillbirth rate data and LBW prevalence data were missing for three and fourteen countries, respectively. Therefore, we were able to assess MRE model fit and performance using 103 countries with complete MMR and stillbirth rate data and using 92 countries with complete LBW prevalence data. MMR and Health Spending did not meet assumptions of normalcy required for analysis. We performed several inverse square root transformations and selected the ones that most effectively reduced skewness and kurtosis. We used the open-source statistical platform R for data analysis [29].

### MRE index scoring characterizations

Developing different scoring characterizations for the MRE Index involved ensuring that very weak or very strong performance on specific elements of the regulatory environment was not masked and deciding how to group and weight the final set of 13 items contributing to the MRE Index [30]. Additionally, we needed to determine whether to use a cumulative or multiple individual components model. The dominant model used in developing composite scores in health research is the *cumulative model*, in which components are combined without considering what specific components they are. This addresses how increasing the total number of regulatory activities in a country impacts outcomes, regardless of their specific activities [31]. This characterization suggests that rather than specific items or domains, it is an accumulation of events that drives outcomes. It answers the question, “What is the impact of increasing the number of regulatory items, regardless of which items they were?” [31].

Several variations of cumulative models are possible to address: 1) how to assign values to individual components for the nine binary items versus two items encompassing multiple questions and two multi-answer option items; 2) different answer options across questions; and, 3) non-ordered answer selections in some cases. This resulted in four scoring characterizations for the MRE Index, shown in Table 2 along with their interpretations and key considerations. Three characterizations (*Any-or-None Scoring*; *Total Sum Scoring*; and *Aggregated Domain Scoring*) treat the MRE Index as a continuous variable, resulting in a single coefficient that describes the expectation of constant change in the outcome as the predictor increases by one unit. The fourth characterization, *Factor Aggregated Domain Scoring*, treats the MRE Index as a categorical variable and generates four separate coefficients to describe the change in outcome as each level of the categorical predictor is compared to a reference level. We also evaluated a multiple individual components model in which individual domains are examined independently in relation to outcomes while controlling for the other domains (i.e., *Domain Scored Results*). This characterization answers the question, “What is the impact on the outcome of the occurrence of each specific domain given the presence/absence of other domains?” [31].

**Table 2:** MRE index scoring characterizations

Scoring type	Characterization	Score range	Interpretation/considerations
**Cumulative Characterizations**			
Any-or-None Scoring	Each of the 13 items comprising the MRE receives 0 or 1 point. Points assigned are no (0)/yes (1); for questions with a larger number of available point values the option is none (0)/any points (1)	0–13	Avoids the inadvertent weighting of any of the multicomponent items by assigning all questions the same point value regardless of answer choices
Total Sum Scoring	Items 1–9 receive no (0)/yes (1) (0–9 possible). Item 10 with 5 answer options receives 0–4 points. Item 11 with 7 questions receives 0 or 1 point for each question (0–7 possible). Item 12 with 5 questions receives 0 or 1 point for each question (0–5 possible). Item 13 with 3 answer options receives 0–2 points	0–27	“Weights” answers for non-binary items, assigning point values for each “yes” answer to questions in multi-component items; Items with more sub-components/questions included in them will contribute more to the score than single component/question items
Aggregated Domain Scoring	The 13 items are aggregated into 5 categories (one for each domain: overarching regulatory policy and legislation; education and qualification; licensure, registration/re-licensure; scope and conduct of practice). A single point for any item in a category (domain) component gets a point for the domain	0–5	Reduces inadvertent weighting toward domains with more items versus domains with fewer items
Factor Aggregated Domain Scoring		0–5	Treats the score as a factor rather than a continuous number, which is possible because of the small score range; can generate coefficients to describe the change in outcome at each level compared to the reference level, allowing each domain to have its own relationship with outcomes rather than assuming a linear relationship
**Multiple Individual Component Characterizations**			
Domain Scored Results	The 13 items are aggregated in 5 categories (one for each domain: overarching regulatory policy and legislation; education and qualification; licensure, registration/re-licensure; scope and conduct of practice). A single point for any item in a category (domain) component gets a point for the domain	0–5	Can produce coefficients for each event separately while controlling for other events in the model; works well for items which are very correlated, as regulatory mechanisms may be

### Model assessment and comparisons

Two descriptive statistics for each model were calculated: *R*^2^ estimates the total variability explained by the model and serves as a summary measure of the model’s predictive power. The Akaike Information Criterion (AIC) assesses how well the model fits the data it was generated from using a log likelihood measure of unexplained information [32].

Model fit was the primary metric used to assess the five composite MRE Index scoring characterizations. The models tested are considered partially non-nested because they used the same covariates but each characterized the MRE Index differently. Formal guidance on model selection is rare in empirical literature, with Vuong [33] and Clarke [34] being the two widely available tests for comparing models. We chose to use Clarke’s test due to evidence suggesting it is more effective with smaller sample sizes [34, 35]. Both Vuong and Clarke first test model *distinguishability* by calculating the ratio of log likelihoods of the models [36]. The Clarke test subsequently applies a modified paired sign test to assess differences in the log-likelihood for each model being compared, determining if the difference is greater or less than zero [35]. All possible pairs of models were compared against each other to gauge their performance relative to each outcome. If the model fit is not significantly different according to the Clarke test, a difference in AIC > 50 can be used to indicate a substantial difference, helping select the ‘best fit’ model [37]. This criterion can be combined with the adjusted R^2^ (predictive performance) to arrive at a final model.

## Results

Descriptive results of the five MRE Index characterizations are presented in Table 3. Bolded values reflect the highest R^2^ and lowest AIC for each model.

**Table 3:** Descriptive results for all midwifery regulatory environment models

Outcome	Maternal mortality ratio^a^		Low birthweight prevalence^b^		Stillbirth rate^a^	
Model type	R^2^	AIC	R^2^	AIC	R^2^	AIC
*Cumulative Models*						
Any-or-None	0.742	580.8731	0.4978	**533.4896**	0.7804	567.9165
Total Sum	0.7281	586.2821	0.4796	536.7545	0.7715	572.028
Aggregated Domain	0.7441	**580.0132**	0.4901	534.8806	**0.7822**	**567.0804**
Factor Aggregated Dmain	**0.7444**	581.6721	0.4921	536.2417	0.7781	570.735
*Multiple Individual Components*	0.7372	586.2446	**0.504**	535.7387	0.7749	573.9442

^a^*N* = 103

^b^*N* = 92

The between-model comparison results are shown in Table 4. For each outcome (MMR, LBW, Stillbirth), the performance of each characterization is compared to each other characterization using Clarke’s test. The model that performed better between the two characterization is listed in the table and statistical significance is noted. Where neither model performed better, “Neither” is used. This table represents only Clarke’s test results; additional statistical tests were needed to determine final characterization selection for each outcome, as described below.

**Table 4:** Preferred models for between-model comparison results for MRE

Outcome	Model	Any-or-none	Total item sum	Aggregated domain	Factor aggregated domain
MMR	Total Item Sum	Neither	-	-	-
	Aggregated Domain	Neither	Aggregated Domain^*^	-	-
	Factor Aggregated Domain	Any-or- None^***^	Total Item Sum^***^	Aggregated Domain^*^	-
	Multiple Individual Components	Any-or- None^***^	Total Item Sum^***^	Aggregated Domain^***^	Factor Aggregated Domain^***^
LBW	Total Item Sum	Any-or- None^**^	-	-	-
	Aggregated Domain	Neither	Aggregated Domain^*^	-	-
	Factor Aggregated Domain	Any-or- None^***^	Total Item Sum^***^	Aggregated Domain^**^	-
	Multiple Individual Components	Any-or- None^***^	Total Item Sum^***^	Aggregated Domain^***^	Factor Aggregated Domain^***^
Stillbirth	Total Item Sum	Any-Or- None^**^	-		
	Aggregated Domain	Aggregated Domain^*^	Aggregated Domain^***^		
	Factor Aggregated Domain	Factor Aggregated Domain^*^	Factor Aggregated Domain^***^	Aggregated Domain^***^	-
	Multiple Individual Components	Any-or- None^***^	Total Item Sum^***^	Aggregated Domain^***^	Factor Aggregated Domain^***^

^*^p  < 0.05; ^**^p  < 0.01; ^***^p  < 0.001

For the MMR outcome, Clarke’s test indicated that while both the Any-or-None and Aggregated Domain Scoring models performed better than all other models, there was no significant preference between the Any-or-None and Aggregated Domain models when compared to each other. We next evaluated AIC and R^2^ to select the best-fit model. Based on this analysis, the Aggregated Domain Scoring characterization (continuous cumulative) emerged as the best fit model with a very slightly lower AIC (− 0.8599 lower AIC) and slightly higher predictive power (0.21% higher than Any-or-None). The Aggregated Domain Scoring model was identified as the best-fit model for the stillbirth outcome as it outperformed other models in all comparisons using Clarke’s test.

For the LBW prevalence outcome, Clarke’s test indicated that the Any-or-None and Aggregated Domain Scoring models outperformed other models, just as we found with the MMR outcome. Ultimately, the Any-or-None model emerged as the best fit model with a very small reduction in AIC (− 1.391 compared to the Aggregated Domain model) and a slight increase in predictive power (0.77% higher compared to the Aggregated Domain model).

The Multiple Individual Components Model demonstrated the poorest performance across all outcomes; it was significantly poorer than all alternative models at a *p* < 0.001 level according to Clarke’s test. Additionally, it had higher AIC values and reduced predictive power compared to most models across all outcomes. Among the cumulative models, the Total Sum Scoring model performed the poorest for the stillbirth outcome based on Clarke’s test, while the Factor Aggregated Domain Scoring model was the poorest performing model for the MMR and LBW prevalence outcomes based on Clarke’s test.

## Discussion

In this research, we developed five characterizations of midwifery regulatory environments in the countries that participated in the Global Midwives’ Associations map survey. We then assessed the fit and performance of each characterization to identify the optimal characterization of the effects of the regulatory environment on three maternal health-related outcomes (MMR, LBW prevalence rate, and stillbirth rate) in order to have an operationalized independent variable (the MRE Index) representing the midwifery regulatory environment construct for future research.

The MRE Index represents a significant step towards better characterization of a construct that represents the complexity of a multicomponent midwifery regulatory environment. The high predictive power of the models we tested indicates that the regulatory environment plays a critical role in maternal health outcomes. This research provides a replicable and transparent methodological process for operationalizing and measuring the MRE. Future research can build on this work by exploring areas such as the impact of the MRE on midwifery recruitment and retention. Overall, this research underscores the importance of midwifery regulation, highlighting its significance for policymakers and encouraging investment in this area.

### Research implications

Our study highlights that a commonly used method among researchers when developing scoring systems [38–40] – simply adding up answers to all questions – can yield some of the poorest results. This was evident in our Total Sum model, where each question contributed equally to an additive score without considering the number of questions in each regulatory domain. Consequently, our Total Sum model disproportionately weighted the scope of practice questions because they had more components rather than considering their inherent significance. While the impact of this weighting was somewhat masked by the small number of items in our survey, on a larger scale, a Total Sum model could significantly inflate scores from multicomponent questions lacking a conceptual basis. An inaccurate, oversimplified, or suboptimal characterization of a predictor variable has significant implications given the reliance on evidence-based research for policy and programmatic decision-making [31]. Incorporating model fit and prediction as standard practice in composite variable development could enhance the reliability and replicability of conclusions derived from research that uses a composite variable predictor.

Expert consensus, often achieved through methods like the Delphi process or modified Delphi process, is a common approach used in developing composite measures. For example, Vedam et al. used a four-round modified Delphi process to identify, rank, and weight items to create a Midwifery Integration Scoring System for assessing midwifery integration in the United States, using regulatory data from multiple databases [41]. While this process is widely used, it has limitations: it can be time-consuming and lack reliability, the definition of “consensus” may be ambiguous, and maintaining ongoing expert engagement can be challenging [42]. Importantly, reaching consensus does not guarantee the ‘correct’ answer has been found; it simply indicates agreement on the importance of certain aspects related to the topic being studied [42]. In an era prioritizing decolonization of global health research, alternative approaches are valuable, especially considering that researchers in LMICs may not have access to a global body of experts [43]. The ‘model fit and prediction’ approach we present offers an excellent alternative for developing a composite variable in many scenarios.

Developing composite measures increases the value of secondary data analysis by effectively and efficiently operationalizing complex health system data. This approach is versatile, cost-effective, and applicable across various outcomes. However, researchers must remain mindful that the methodology used to develop final composite measures may significantly affect research findings in terms of reliability, validity, and usefulness [18, 44]. Therefore, transparent and replicable approaches are essential, along with justification of the methods used [45]. Our study demonstrates how this methodology can be used to create a composite variable for subsequent use in studies with significant global health implications and offers a framework for other researchers to undertake similar processes. When regulatory data is not available for a health profession, these findings demonstrate the value of routine regulatory data collection and how this data can be used to strengthen regulatory efforts.

### Scoring characterizations and outcomes

Our results also indicate that different scoring characterizations may be preferred for different outcomes, a finding that has important implications for interpreting relationships between midwifery regulatory environment and outcomes. The Aggregated Domain model (which assigns a single point for at least one category in which action is being taken in each domain) was the best fit for both MMR and stillbirth rate. This suggests that no single domain is more important than another and that even limited activities within a domain can significantly strengthen the regulatory environment. The Aggregated Domain model is relatively straightforward to understand and calculate, aiding replicability and preventing inadvertent weighting of certain domains due to some domains having more questions than others. Overall, this model suggests that countries making efforts to address regulation across all domains may achieve better outcomes than those focusing more heavily on one area while neglecting others. Additional research is needed to confirm how this finding is reflected in practice across different countries.

The best model fit and performance for the LBW prevalence outcome was the Any-or-None model. This may be because prevalence of low birth weight is, in many ways, a more complex and multifactorial phenomenon than stillbirth rate and MMR. While intrapartum stillbirths, which account for approximately half of all stillbirths globally [46] and as much as 75% of stillbirths in some countries [47], and MMR are heavily dependent on care provided during the intrapartum period, birthweight is already determined by the time a patient presents for intrapartum care, except in cases of premature labor where skilled care can sometimes delay birth. LBW is multi-factorial with contributing factors including maternal nutrition before and during pregnancy, antenatal care attendance, acute (e.g., malaria, syphilis) and chronic maternal health conditions (e.g., hypertension), and lifestyle choices (e.g., smoking) [48]. Thus, while LBW prevalence is a sensitive indicator of maternal health care quality, it indicates the quality of care across the reproductive lifespan, and the variation in the best fit model may reflect this. Comparing model fit and performance between fresh stillbirth (no sign of maceration) outcomes, which are more likely related to intrapartum care, and macerated stillbirth outcomes, which are more likely to have multifactorial antepartum causes similar to LBW, might provide additional insights into why one midwifery regulatory environment model works better for intrapartum-focused outcomes and another for antepartum or reproductive lifespan outcomes.

### Limitations

This study was intended to compare different characterizations of the MRE predictor variable—not the effects of regulatory environment on outcomes—and so we do not report here the specific individual effect estimates of the MRE on outcomes. In order to compare models using Clarke’s test, all models included the same covariates, regardless of collinearity with the composite measure or each other. This ensured that differences between models were due to differences in characterization of the MRE Index, because variation from the covariates remains constant across models. These decisions do affect model outcomes and how models should be interpreted. Additionally, we did not use a multi-level modeling approach in this study because of data limitations, and so we are not able to detect differences in the correlates of MMR that may differ by country. Additional research on this topic would be a valuable contribution.

## Conclusions

Midwifery regulation is widely acknowledged as crucial for effective midwifery care, which significantly impacts maternal health outcomes. Our study demonstrates that using model fit prediction with existing datasets offers a transparent and accessible approach for developing a Midwifery Regulatory Environment (MRE) Index with strong predictive power for key maternal health outcomes (MMR, LBW prevalence, and stillbirth rate). The MRE Index can be a valuable tool for researchers investigating relationships between regulatory environments, midwifery-related factors, and health outcomes. The broader takeaway from this study is that global health researchers should consider integrating model fit prediction in developing composite measures during secondary data analysis. This can enhance the transparency, replicability, and accuracy of their findings, providing valuable insights for policymakers.

## Supplementary Information

Supplementary Material 1.

## Data Availability

The dataset was compiled from publicly available sources. The compiled dataset used in data analysis during the current study is available from the corresponding author on reasonable request.

## Abbreviations

AIC: *Akaike* Information criterion
BEmONC: Basic Emergency Obstetric and Newborn Care
ES: Education sub-section
HDI: Human Development Index
ICM: International Confederation of Midwives
LBW: Low birthweight
LMIC: Low and middle-income countries
MMR: Maternal mortality ratio
MRE: Midwifery Regulatory Environment
RS: Regulatory sub-section
UNDP: United Nations Development Program
UNICEF: United Nations Children’s Fund
UNFPA: United Nations Population Fund
WHO: World Health Organization
