# Supplementary material for: Development of a midwifery regulatory environment index using data from the Global Midwives’ Associations map survey
Source: BMC Health Serv Res. 2025 May 20;25:728. doi: 10.1186/s12913-025-12694-w (PMC12093735; doi:10.1186/s12913-025-12694-w)
Supplement: Supplementary file 1 — Supplementary Material 1. [file 12913_2025_12694_MOESM1_ESM.docx]

| **ICM Question Number** | **Question** | **Answer Choices** | **Included/Excluded and Reason for Exclusion** |
| --- | --- | --- | --- |
| Regulation Sub-Survey (RS) 1 | Name of the Midwives Association(s) | Free-text | Excluded; free-text for the purposes of providing clarifying information not directly relevant to a regulatory domain |
| RS2 | Country | Free-text | Technically included but used as identifying information and not part of regulatory domain scoring |
| RS3 | Is there legislation in your country which recognizes midwifery as a profession that is distinct from nursing? | Y/N | Included |
| RS4 | Is there legislation through which midwives are regulated? | Y/N | Included |
| RS5 | What is the name of the legislation or title of the Act? | Free-text | Excluded; free-text for the purposes of providing clarifying information not directly relevant to a regulatory domain |
| RS6 | Is there a regulation system for midwives in your country? | Y/N | Included |
| RS7 | What is the name of the Regulatory Authority? | Free-text | Excluded; free-text for the purposes of providing clarifying information not directly relevant to a regulatory domain |
| RS8 | What type of organization is the Regulatory Authority? | Government department/ Government-approved board/ Council / other | Excluded; free-text for the purposes of providing clarifying information not directly relevant to a regulatory domain |
| RS9 | What are the key functions of the Regulatory Authority? | Free-text | Excluded; variety of free-text responses made categorization difficult |
| RS10 | Does the same regulatory authority regulate other health professionals as well as midwives? | Y/N | Excluded; information relevant for assessing regulatory environment captured in question RS12 |
| RS11 | Which other health professions are regulated in the country? | Free-text | Excluded; provides contextual information but not directly relevant to assessing a country’s regulatory domain and free-text answers varied wildly |
| RS12 | If the regulatory authority regulates midwives and other professions, are there separate and distinct policies and processes? | Y/N | Included |
| RS13 | What term do you use to describe the legal right to practice and to use the title of midwife? | Licensing/ Registration/Both licensing and registration as separate and mandatory processes/Other | Excluded; information captured more directly in other questions |
| RS14 | Please enter a link to the governmental website(s) used to collect the above data *(if not applicable put NA*) | Free-text | Excluded; free-text for the purposes of providing clarifying information to directly relevant to a regulatory domain |
| RS15 | Is there a system for licensing midwives? | Y/N | Included |
| RS16 | Is licensing compulsory before midwives start to practice in the country? | Y/N | Included |
| RS17 | Are midwives required to relicense in order to practice?^a^ | Y/N | Included |
| RS18 | As a condition of re-licensing, are midwives required to provide evidence that they have participated in continuing professional development activities? | Y/N | Included |
| RS19 | 19. Please enter a link to the governmental website(s) used to collect the above data *(if not applicable put NA*) | Free-text | Excluded; free-text for the purposes of providing clarifying information to directly relevant to a regulatory domain |
| RS20 | Is registration required to practice midwifery in the country? | Y/N | Included |
| RS20a | What is the name of the organization that registers midwives? | Free-text | Excluded; free-text for the purposes of providing clarifying information not directly relevant to a regulatory domain |
| RS20b | Please enter a link to the governmental website(s) used to collect the above data *(if not applicable put NA*) | Free-text | Excluded; free-text for the purposes of providing clarifying information not directly relevant to a regulatory domain |
| RS21 | How often does this organization update the register to add new midwives or remove those who have left the profession? | Daily/ Monthly/ Several times a year)/ Annually/ Less Often^b^ | Included |
| RS22 | Number of midwifery graduates employed in the last year | Numeric | Excluded; not directly relevant to one of the five regulatory domains |
| RS23 | Number of midwifery graduates registered in the last year | Numeric | Excluded; not directly relevant to one of the five regulatory domains |
| RS24 | Total number of registered midwives in the country *(i.e. total number of practicing and non-practicing midwives*) | Numeric | Excluded; not directly relevant to one of the five regulatory domains |
| RS25 | How many of the registered midwives are practicing? | Numeric | Excluded; not directly relevant to one of the five regulatory domains |
| RS26 | How many of the registered midwives are non-practicing? | Numeric | Excluded; not directly relevant to one of the five regulatory domains |
| RS27-RS33 | Are midwives authorized to provide this BEmONC function:   - parenteral administration of antibiotics - administration of anticonvulsants - administration of oxytocics - manual removal of placenta - manual vacuum aspiration for retained products - assisted instrumental delivery by vacuum extractor - newborn resuscitation with mask | Y/N  Y/N  Y/N  Y/N  Y/N  Y/N  Y/N | Included |
| RS34-RS38 | Are midwives in this country authorized to provide:   - contraceptive injection - contraceptive pill - intrauterine device - emergency contraception (morning after pill) - contraceptive implant | Y/N  Y/N  Y/N  Y/N  Y/N | Included |
| RS39 | Please enter a link to the governmental website(s) used to collect the above data *(if not applicable put NA*) | Freetext | Excluded; free-text for the purposes of providing clarifying information not directly relevant to a regulatory domain |
| Education Sub-Survey (ES) 4 | Does your country have a national curriculum for midwifery education, whether is direct entry or post-nursing? | Yes, and all schools follow it /Yes, and some schools follow it/No^c^ | Included |
